# Quality of life among parents of children with heart disease

**DOI:** 10.1186/1477-7525-6-91

**Published:** 2008-11-03

**Authors:** Mostafa A Arafa, Salah R Zaher, Amira A El-Dowaty, Dalia E Moneeb

**Affiliations:** 1 Epidemiology Department, High Institute of Public Health, Alexandria University, Alexandria, Egypt; 2 Pediatric Department, Faculty of Medicine, Alexandria University, Alexandria, Egypt; 3 Mental Health Department, High Institute of Public Health, Alexandria University, Alexandria, Egypt; 4 Fellow of Epidemiology, High Institute of Public Health, Alexandria University, Alexandria, Egypt

## Abstract

**Background:**

Quality of life of parents of chronically ill children has become increasingly important as the mortality rates associated with such illnesses have decreased and survival rates have increased.

**Aim:**

To describe the Health related quality of life (HRQOL) of parents whose children are suffering from heart diseases and to identify the most important factors that could affect it.

**Methods:**

A cross sectional study was conducted in Alexandria, Egypt in the two main hospitals that treat children with heart diseases. 400 parents of children with heart diseases were recruited and a comparison group (400) of parents of children with minor illnesses were included from both hospitals. Socioeconomic and disease related data were collected, SF36 was used to collect data regarding the QOL. MANOVA was used to compare the SF-36 scores between groups and to explore the impact of different variables.

**Results:**

In all SF-36 subscales, parents of children with heart diseases reported significantly poorer HRQOL, except for pain subscale. The most striking differences were for General Health, Vitality and role limitation physical. Factors that had a significant impact of HRQOL were severity of illness, type of heart disease in addition to age of child, having multiple children, financial situation and presence of comorbid condition. The mean scores for different domains were the lowest for younger age, rheumatic heart disease and female children.

**Conclusion:**

QOL of parents of children with heart diseases was significantly impaired and it was influenced by several factors; mainly related to the clinical status of the child. Psychological status, social support and reassurance of the parents should be considered when making treatment decision for their children.

## Background

The incidence of congenital Heart Diseases (CHD) was reported to be 8/1000 live born infants [1]. Annually, there are 400,000 deaths and hundred of thousands of children died due to rheumatic fever (RF) and rheumatic heart diseases[2]. The prevalence of heart diseases in children in Egypt is not precisely estimated. moreover, the incidence of RF is not expected to dramatically decline in the near future. Quality of Life (QOL) is an estimate of remaining life free of impairment, disability or handicap[3]. Chronic conditions put increased stress on the child and the child's parents and siblings. Children with any chronic condition have twice the risk of developing mental health disorders of healthy children, and three times the risk if they have an accompanying disability[4]. The parents rate their children's QOL to be worse than the children themselves do[5]. It may be affected by their expectations for the child and by the fact they have different definitions and understanding of a disease and its consequences for the future [6-8].

Although it has been argued that all chronic illnesses can negatively affect health-related quality of life of the parents of disabled children; each disease present unique challenges,[9] for example, parents of asthmatic children-have poor QOL especially in their emotional domain affecting their social life[10]. Regarding parents of cancer children, it was found that they have high levels of anxiety and depression especially in the months immediately after diagnosis of cancer[11]. Studies of parents of epileptic child have showed that their major concern was regarding the child's seizures, loss of consciousness, ill effects of anti-epileptic drugs and their only major concern in family life was that having an epileptic child put an added strain on their marital relationship[12].

The finding on QOL among parents of disabled children are contradictory, in addition little is known about the identification and quantification of determinants of QOL among parents of children with heart diseases. The number of studies conducted are very scarce and non of them was carried out on a developing country like Egypt. Hence, this work was undertaken to describe the QOL of parents of children with heart diseases and to identify the most important factors that had an impact on it.

## Methods

A comparative cross-sectional design was used in the heart clinics of the University Children's Hospital (El-Shatby) and Student's Health Insurance Hospital in Alexandria, Egypt and the E.N.T, surgery and dehydration clinics of the same hospitals during the period February through July 2007. Any cardiac patient irrespective of his age or residential origin can attend these clinics for assessment, follow up and management. Our target population included two groups; the parents of the children with heart disease who were diagnosed as cardiac patients (congenital or rheumatic heart disease) and they amounted to 400 and a comparative equal number from the parents accompanying their children who were attending the outpatient clinics for minor illnesses (upper respiratory tract infection, sore throat, abscess, and diarrheal diseases). The cardiac clinics for each hospital receive their patients only two days a week; during which all parents attending these clinics with their children during the period of study were included in our sample. For each cardiac case, a parent of non cardiac child was selected randomly. An informed and verbal consent was obtained from parents before the interview. Study participants (only one parent) were interviewed in person by trained interviewers using a structured questionnaire to elicit information on the current; socio-demographic characteristics, heart disease related data (type, severity, onset of diagnosis and treatment, compliance, any accompanying morbidity), family related risk data (effect of the disease on the expenses and spending, how much parents are concerned for the professional and health future of their diseased child and their expectations). Severity of heart disease was checked from the attending physician and confirmed from the records, it depended on the symptoms, presence of cardiomegally and pulmonary hypertension, and the severity of valvular lesions. Financial situation was elicited by asking parents of cardiac diseased children whether they are complaining of financial problems because of the increased expenses on their diseased child. The studies' committee for research ethics at the high institute of Public Health approved the study.

### Health-related quality of life

The SF-36 V1, which serves as the generic core of the QOLIE-89 [13], the translated Arabic form was used to evaluate HRQL[14]. This 36-item measure is made up of eight subscales, each evaluating a different domain of HRQL: physical functioning, physical role, bodily pain, general health, vitality, social functioning, emotional role, and mental health. Subscale scores are calculated according to standard procedures, yielding score values of 0 to 100; higher scores indicate better quality of life. The eight subscales can be aggregated into the two composite indices (Z scores): physical component summary (PCS) score and mental component summary (MCS) score, which utilize population means and standard deviation to establish the scores. All PCS/MCS are norm based with the general population means equal to 50 and the standard deviation equal to 10.

#### Analysis

Results were expressed as frequencies, means and standard deviations. SF-36 subscale scores for the participants in this study were compared across the two groups of study using multivariate analysis of variance (MANOVA). MANOVA was used also to explore the impact of the socio-demographic, medical and family related factors on the QOL among parents of the children with heart disease. The alpha level for MANOVA test was set at 0.05. Significant statistics (p < 0.05) were followed by post hoc analyses to determine which subscale were showing group differences, and which specific groups were significantly different from one another. The two composite indices; Physical component summary and mental component summary score (PCS & MCS) could not be obtained as we don't have the measurements for population norms for the QOL domains in our culture.

## Results

### 1 – General characteristics of the study sample

The total number of the studied cases amounted 800 parents, only 3 refused to participate, their description is illustrated in table 1. Their ages ranged 20 – 58 years More than half of the sample were just read and write, those with secondary and university education represented 30% and 32% for both groups. More than 90% of our sample were married house wives. More than two thirds of the families (71%) were of lower and middle social class. As regards children; their age ranged from 1 month to 202 months with a mean of 68.5 ± 61.2. There were excess females in the heart diseased group. Nearly two thirds of children (67.5%) had congenital heart disease and the rest (32.5%) had rheumatic heart disease. Those suffered from severe form of illness constituted the least figure(7.8%) followed by mild degrees (28.7%), while the highest figure (63.5%) was among those with moderate degree of heart disease. Nearly 13% of heart diseased children (51 cases) were complained of accompanied co-morbidity in the form of Rheumatoid arthritis, upper respiratory tract infections and Down syndrome. It is worth mentioning that 98% of the parents of heart diseased children expressed their concern about future familial, financial and health adjustment problems of their children. More than two thirds (70%) complained of financial problems because of increased expenses as a result of presence of a disabled child.

**Table 1:** General characteristics of the study sample

	Parents of children with Heart disease	Parents of children with minor illness
**Age**	Mean & SD, 35.7 ± 20.4	Mean & SD, 34.7 ± 19.8

**Education**		
Read and write	256, 64.0%	213, 53.2%
Primary & preparatory	25, 6.3%	59, 14.7%
Secondary and higher	119, 29.7%	128, 32%

**Marital status**		
Married	378, 94.5%	393, 98.25%
Divorced	3, 0.7%	1, 0.25%
Widowed	19, 4.8%	6, 1.5%

**Occupation for Fathers**		
Manual and trade	309, 77.3%	336, 84%
Employee	88, 22%	64, 16%
Out of work	3, 0.7	------
**Occupation for Mothers**		
House wives	381, 95.2%	392, 98%
working	19, 4.8%	8, 2%

	**Children with Heart diseases**	**Children with minor illnesses**

**Age**	Mean & SD, 73.2 ± 64.3	Mean & SD, 63.8 ± 57.6

**Sex**		
Males	197, 49%	209, 52.3%
Females	203, 50.7%	191, 47.7%

**Grade**		
Preschool	226, 56.6%	256, 64%
Primary	115, 28.7%	105, 26.3%
Preparatory	37, 9.3%	22, 5.5%
Secondary	22, 5.5%	17, 4.2

**Type of disease**	CHD 270, 67.5%	RTI 256, 64%
	RHD 130, 32.5%	Diarrhea 133, 33.3%
		Abscesses 11, 2.7%

CHD Congenital heart disease RHD Rheumatic Heart disease

RTI respiratory tract infection

### 2 – Health related quality of life: SF-36 profile

Table 2 revealed that parents of the children with heart disease had significantly worse mean scores in all HRQOL dimensions when compared to data from parents of children with minor illnesses, except for the bodily pain subscale. The most striking differences were observed in the vitality subscale (39.66 vs. 75.81) general health (46.25 vs. 73.15) and role physical (39.53 vs. 61.81). The lowest differences although significant were seen for the physical functioning (75.76 vs. 79.84) and social functioning (93.63 vs. 99.88). The overall test statistic was statistically significant (P < 0.001) for the seven subscales indicating that there was a relationship between group membership (parents of children with heart disease and parents of children with minor illnesses) and HRQOL. MANOVA indicated a significant impact of age (F = 6.77, p < 0.001), sex (F = 2.191, p < 0.05), type of heart disease (F = 5.197, p < 0.001), severity of illness (F = 5.848, p < 0.001), the presence of co-morbid conditions (F = 3.782, p < 0.001), financial situation of the parents (F = 5.880, p < 0.001), and number of children (F = 2.243, p < 0.005) on HRQL of parents of children with heart disease. Education and sex of the accompanying person of the child were not statistically significant (F = 1.072, p = 0.357) and (F = 1.33, P = 0.380) respectively, table 3. Eta square was also presented in that table which describes the proportion of total variability attributable to a factor, Table 3.

**Table 2:** Comparison of SF-36 subscales between groups

SF-36 subscales	Group	$\bar{\text{X}}$	SD	F statistic	P-value
General health	Cases	46.25	23.59	277.87	0.000
	Controls	73.15	22.03		

Physical functioning	Cases	75.76	17.11	13.51	0.000
	Controls	79.84	14.10		

Role limitation due to physical health problems	Cases	39.53	34.35	71.67	0.000
	Controls	61.81	39.89		

Role limitation due to emotional problems	Cases	38.25	32.46	69.32	0.000
	Controls	58.75	37.03		

Vitality (Energy/fatigue)	Cases	39.66	23.71	480.42	0.000
	Controls	75.81	22.94		

Emotional well-being	Cases	72.90	16.71	75.24	0.000
	Controls	82.67	15.12		

Social functioning	Cases	93.63	21.31	33.93	0.000
	Controls	99.88	2.50		

Bodily pain	Cases	82.60	11.6	3.57	0.344
	Controls	81.80	12.3		

**Table 3:** MANOVA general F-test, Factors affecting HRQL.

Factor	F statistic	P-value	Eta squared
Age	6.776	0.000	0.10

Sex	2.191	0.034	0.041

Type of heart disease	5.197	0.000	0.11

Severity of illness	5.848	0.000	0.12

Presence of co-morbid condition (Down syndrome, rheumatic arthritis)	3.782	0.001	0.05

Financial situation	5.880	0.000	0.046

Education of the parents	1.072	0.357	0.017

Parental sex	1.33	0.380	0.015

Number of children	2.243	0.006	0.37

The results of the univariate analysis are shown in table 4. Age of the child was associated significantly with poorer HRQOL in the areas of role physical (F = 7.814, P < 0.05) and role emotional (F = 15.389, P < 0.05), while child ser was associated with poorer HRQOL only in areas of vitality (F = 5.036, P < 0.05) and social functioning (F = 4.832, P < 0.05). Notably, severity of illness had a significant impact on poorer HRQOL in all QOL domains, also type of heart disease was associated with poorer HRQOL in all areas of the QOL domains except social functioning and emotional well-being. Financial situation of the parents and associated co-morbid conditions were associated with significantly poorer HRQOL in areas vitality and emotional well-being. Physical and social functioning subscales were affected also by the financing problems of the parents (F = 8.821, P < 0.05 & F = 13.734, P < 0.001 respectively). The only domain that sound to be affected significantly by number of children was the social functioning. Parents QOL was worse for female children with rheumatic heart disease and with severe degrees of the disease for the corresponding significant domains. With regards to child age, it was categorized into 3 categories, less than 6 years, 6–13 years and more than 13 years. It was noticed that the mean score for QOL was the least for the lowest age category, it increased to the highest in the primary school age category, then during the adolescence (> 12 years) it worsens again.

**Table 4:** MANOVA Univariate test.

QOL Domain	Factor	F statistic	P-value
Role physical^c^	Child's age	7.814	0.005
Role emotional^d^		15.389	0.000

Vitality ^e^	Child's sex	5.036	0.025
Social functionin^g^		4.832	0.029

General health^a^	Type of heart disease	3.962	0.047
Physical functioning^b^		8.540	0.004
Role physical ^c^		5.951	0.015
Role emotional^d^		9.945	0.002
Vitalit^e^		3.601	0.044

General health^a^	Severity of illness	19.702	0.000
Physical functioning^b^		7.156	0.001
Role physical^c^		14.047	0.000
Role emotional^d^		8.926	0.000
Vitality^e^		14.867	0.000
Emotional well-being^f^		19.817	0.000
Social functioning^g^		14.435	0.000

Vitality^e^	Presence of co-morbid condition	4.443	0.036
Emotional well-being^f^		15.890	0.000

Physical functioning^b^	Financial situation	8.821	0.003
Vitality^e^		15.950	0.000
Emotional well-being^f^		6.918	0.009
Social functioning^g^		13.734	0.000

Social functioning^g^	Number of children	3.579	0.029

Influence of significant variables on HRQL domains.

a R^2 ^= 0.11, b R^2 ^= 0.53, c R^2 ^= 0.72,

d R^2 ^= 0.63, e R^2 ^= 0.13, f R^2 ^= 0.15

g R^2 ^= 0.08

## Discussion

Heart disease is rated among the most severe of chronic disabilities among children. Parents of the children with heart disease may experience higher stress levels than parents of children with other diseases, and may feel great stress in relation to such things as dilemmas of normality and social integration [15,16] On the other hand the parents' QOL may be more determined by spouses' satisfaction with each other or parental coping styles than by the child's handicap [17-19].

Results of this study support the premise that parents of the children with minor illness experienced a better HRQOL than those of the children with heart disease. Parents of the children with heart disease reported severe impairment across multiple domains of QOL including a lowered sense of well-being with regard to energy and general health, limitations in function due to physical and emotional reasons. The greatest mean differences between parents were found on the vitality, general health and role limitation-physical scales. As these domains deal with parents' feeling of physical health including energy and fatigue, problems of work or other daily activities which resulting from physical health. These findings were in agreement with the study carried out among Swedish population which addressed the problems of parents of the children with heart disease in different areas of the QOL. It indicated that parents of the children with heart disease had significantly poorer QOL than parents of the children with other diseases and those of healthy children[20].

parents of the children with heart disease expressed that caring load reduced their time/opportunity to unwind, interfered with family activities and family cohesion, as well as recreation and socialization activities which in turn may have negatively affected their QOL. Parents of children with developmental disabilities showed that parenting these children is associated with impaired mental health, higher levels of stress, and also impaired physical functioning [21-24]. These parents accommodated to their child's needs early on by restricting their social life and making changes in family routines.

More than 90% of the interviewed parents were the mothers which might explain the result of the present study that parental sex did not account for the reduced QOL. In contrary to the studies of Lawoko S, Wysocki T and Wikblad K [20,25,26] which indicated that mothers of children with heart disease reported lower QOL than fathers with a particularly great impact on the social, physical and psychological subscales. As well, QOL of mothers of children newly diagnosed with cancers was much worse than fathers as compared to general population. This may be because mothers tend to be more involved than fathers in care-giving and spent extra time caring for their sick child, more responsible for medication and treatment decisions, and more likely to stay in hospital with the child.

One of the aims of the present study was to consider the most important factors which may be involved in the effect of heart disease on the QOL of the parents. The present study revealed that severity of illness, type of heart disease and financial status of the family were associated significantly with poorer HRQOL in nearly most of the subscales; particularly severity of illness where all domains were affected. Whereas having multiple children had an influence only on social functioning. These factors tend to lay additional physical, emotional and financial burden on the family reducing their QOL. This was evidenced by the impairment in the physical functioning, vitality, general health, and role physical domains for those parents as these domains deal with all physical activities including household activities, bathing, dressing, energy and fatigue. On the other hand the study of Lawoko S [20] indicated that the reduced QOL was more determined by such variables as financial burdens, distress & hopelessness than by the child's sex and illness.

In accordance with our findings which showed a great impact of the financial situation of the parents on the physical functioning, vitality, emotional well-being, and social functioning scales of the QOL, a study of a Swedish population showed that parents who were concerned about their finances and with living expenses problems expressed lower QOL than those without such difficulties [20].

SF-36 was used several times, among different patients and in many different cultures, however this was the first time to use this tool in our developing region for examining the QOL of parents of cardiac diseased children particularly rheumatic heart disease which is not a feature of comparables studies and so is a novel area, which could enhance and strengthen the outcome of the current work.

### Limitations of the study

Our data is representing the children population with heart diseases in our community as most of such diseased children are managed in that governmental clinics and nearly all of them are of the same social class. However, the points of the weakness should be acknowledged. Firstly, our data depended entirely on the parents' subjective assessment of their own QOL which may question its accuracy. Secondly, the group of children with minor illnesses are less homogenous and their clinical status are much less severe in comparison to heart diseased children. In addition to that, the psychological and health status of parents of heart diseased children were not addressed which might have an effect on their QOL. Also most of the factors that affecting the QOL were non modifiable (child age and sex, financial problems, type of disease, and severity).

### Implications for practice and research

Our study provides preliminary implications for interventions aimed at improving QOL among parents of children with heart disease as the family has the primary and the most important role in the management of such diseases in children. Such interventions could be in the form of social support, Psycho-education of parents and other family members which should go hand in hand with that of children. Besides educating them issues related to their illness, developing their parenting skills and their coping with stress of having a chronically ill child should be emphasized. Being the first study that was conducted in our region about HRQOL of health persons; additional research should be directed at the parents' QOL and the factors improving or worsening it.

## Abbreviations

CHD: Congenital heart disease; RHD: Rheumatic heart disease; RTI: Respiratory tract infection; RF: Rheumatic fever; QOL: Quality of Life; PCS: Physical component score; MCS: Mental component score

## Competing interests

The authors declare that they have no competing interests.

## Authors' contributions

MA conceived of the study, and participated in its design and coordination. SZ supervising the field work, and participated in writing the paper. AE participated in data collection. DE participated in the sequence alignment. All authors read and approved the final manuscript.
